# Editorial: Needs and potential application of One Health approach in the control of vector-borne and zoonotic infectious disease

**DOI:** 10.3389/fmicb.2022.1089174

**Published:** 2022-11-22

**Authors:** Xinyu Feng, Sibao Wang, Gong Cheng, Xiaokui Guo, Xiaonong Zhou

**Affiliations:** ^1^National Institute of Parasitic Diseases, Chinese Center for Disease Control and Prevention (Chinese Center for Tropical Diseases Research), Beijing, China; ^2^National Health Commission (NHC) Key Laboratory of Parasite and Vector Biology, Beijing, China; ^3^World Health Organization (WHO) Collaborating Centre for Tropical Diseases, Beijing, China; ^4^National Center for International Research on Tropical Diseases, Beijing, China; ^5^School of Global Health, Chinese Center for Tropical Diseases Research, Shanghai Jiao Tong University School of Medicine, Shanghai, China; ^6^One Health Center, Shanghai Jiao Tong University-The University of Edinburgh, Shanghai, China; ^7^Department of Biology, College of Life Sciences, Inner Mongolia University, Hohhot, China; ^8^Chinese Academy of Sciences (CAS) Key Laboratory of Insect Developmental and Evolutionary Biology, CAS Center for Excellence in Molecular Plant Sciences, Institute of Plant Physiology and Ecology, Chinese Academy of Sciences, Shanghai, China; ^9^CAS Center for Excellence in Biotic Interactions, University of Chinese Academy of Sciences, Beijing, China; ^10^Tsinghua-Peking Center for Life Sciences, School of Medicine, Tsinghua University, Beijing, China

**Keywords:** One Health, global health, microbiology, vector-borne disease, zoonotic disease

In view of the unbridled outbreaks and increasing prevalence of zoonotic diseases and other infectious diseases, the global health community advocates adopting the “One Health” approach to prevent and cope with these challenges (Jones et al., 2008; Keusch et al., 2022). Some countries have adopted relevant strategies in the campaign against zoonotic diseases and achieved some initial success (Bird and Mazet, 2018; Acharya et al., 2020). For example, from 2013 to 2019, Tamil Nadu in India established a “One Health” committee to address the challenge of dog and human rabies. Finally, the intervention measures developed by the committee reduced the human rabies mortality rate economically and effectively (Fitzpatrick et al., 2016; Gibson et al., 2022). Also, integrated measures against echinococcosis based on the concept of One Health were deployed in Shiqu County, Sichuan Province, China, which significantly reduced the prevalence of echinococcosis after long-term monitoring (Tiaoying et al., 2005; Wang et al., 2021). In addition, since 2009, Switzerland, New Zealand, and other countries have built campylobacteriosis surveillance and management systems by implementing the “One Health” strategy, and the campylobacteriosis epidemic has been effectively contained (Babo Martins et al., 2017; Schiaffino et al., 2019). All the evidence showed that the application of the “One Health” strategy in controlling infectious diseases and zoonoses has achieved good results and led to substantially more significant socio-economic benefits worldwide (Ajuwon et al., 2021). Therefore, this topic discussed the needs and potential application of the One Health approach in practice, program, and policy of vector-borne and zoonotic infectious diseases.

Zoonotic and vector-borne diseases contribute significantly to the global burden of diseases. For instance, malaria, the most important vector-borne disease, affects millions of people and contributes significantly to the disadvantage of public health and socioeconomic development. In this Research Topic, Kassegne et al. characterized different species of plasmodium parasites (*Plasmodium ovale* and *Plasmodium malaria*), which were not previously reported, in high-transmission areas of southern Togo of tropical Africa. The molecular survey of malaria infections in the area helped reveal the natural malaria's epidemiological status. It provided helpful information to improve disease control/surveillance strategies and policies in such areas of endemic malaria. Besides, Rift Valley fever virus (RVFV) is a mosquito-borne viral zoonosis causing severe disease in humans and ruminants. Although the disease is classified as a priority disease by the World Health Organization, licensed vaccines are presently unavailable or contraindicated. Zhang et al. constructed a bacterium-like particle vaccine (BLP), RVFV-BLPs. They also determined that mice immunized with RVFV-BLPs produced both humoral and cellular immunity. RVFV-BLPs represent a novel and promising vaccine candidate for the prevention of RVF infection in both humans and veterinary animals. The described RVFV-BLPs were also found to have the advantage of large-scale use and relatively low cost. In addition, the Japanese encephalitis virus (JEV) is one of the most important emerging pathogens, which causes not only fatal neurological disease in humans but also causes reproductive failure in pigs. By comparing pathogenicity between Japanese encephalitis virus strains (SA14 and BJB), Xing et al. found that the SL-IV and DB1 regions of 3′UTR were essential for JEV replication, neural invasiveness, and viral pathogenicity. They confirmed that some mutations at sites 248, 254, 258, and 307 in the 3′UTR of JEV play a vital role in the viral life cycle. This study offered a new perspective for designing and formulating a candidate vaccine.

Increasing environmental change, global migration, acts of bioterrorism, and human social behavior change may increase the risk of pathogen spill over, such as from wildlife reservoirs to humans, from abundant domestic reservoir hosts to the susceptible animal populations, and from living organisms to the environment and vice versa. More comprehensive prevention and control strategies and meaningful risk management tools are essential to reduce disease spillovers and prevent disease emergence. Olaya-Galán et al. evaluated a zoonotic infection of bovine leukemia virus (BLV) in human beings by gathering experimental evidence about the susceptibility of human cell lines to BLV infection. Several human cell lines (iSLK and MCF7) produced a stable infection throughout the 3 months, supporting the hypothesis of a natural transmission from cattle to humans. This study provided *in vitro* experimental evidence of BLV as an exogenous etiological agent of human breast cancer.

Previous research has reported non-human primates (NHPs) as reservoirs for human intestinal protozoa infection. Li J. et al. investigated the prevalence of pathogenic intestinal protozoan infections in macaques and humans and conducted a risk evaluation of interspecies transmission among laboratory macaques, animal facility workers, and nearby villagers from One Health Perspective. They found that the facility workers had direct contact with macaques and had a significantly higher intestinal protozoa infection rate. Furthermore, some shared haplotypes confirmed the presence of zoonotic subtypes in NHPs and humans. These results warrant the utility of One Health frameworks to characterize infection risk and to offer relevant and comprehensive control strategies in the future.

Microbiological hazards form a major source of food-borne diseases in humans. Solís et al. reported that pet food, especially new feeding practices, such as raw meat-based diets, can be a potential source of microbiological hazards that might affect companion animals and owners. Therefore, microbiological hazards of foodborne pathogens in raw and extruded canine diets may facilitate the causative agent spillover by transmission from a reservoir population, which implies a significant concern for humans and pets.

Emerging infectious diseases (EID) have rapidly increased in recent years and expanded in geographic range. Many EIDs (~60%) are zoonotic, including HIV-AIDS, Ebola and SARS, and COVID-19. Yeo et al. investigated the prevalence of emerging or re-emerging human enteric viruses in porcine stools and swabs in the Republic of Korea. The study demonstrated that human enteric viruses detected in pigs and some porcine enteric viruses are genetically related to human enteric viruses, indicating the zoonotic potential of porcine enteric viruses as potential EIDs.

Generally, influenza A viruses (IAVs) infections are refractory to mammals because of species barriers. On rare occasions, however, IAVs can break the species barrier and spill over to mammalian species. Except for a small number of viruses known to infect animals, including swine, bats, and humans, the emergence of H3N2 canine influenza viruses (CIVs) provides a new perspective for interspecies transmission of the virus. Given this, Li X. et al. screened for amino acid transitions involved in adapting IAVs to canine and other mammalian hosts. They found that the H3N2 influenza virus has host-adaptive signatures in canines and can establish persistent transmission in lower mammals. All these studies highlight the necessity of identifying and monitoring the emerging pathogen spillover effects by enhanced surveillance and further studies to ensure an integrated “One Health” approach that aims to balance and optimize the health of humans, animals, and ecosystems.

Timely and sensitive detection is particularly important for the detection of potential pathogens. Molecular methods offer improved sensitivity for detecting pathogens through a diverse context. Herein, Yao et al. developed a cost-effective, multi pathogen and high-throughput method for simultaneously detecting the Ebola virus and 16 other pathogens associated with hemorrhagic fever. The simultaneous detection assay would provide a reliable and sensitive diagnostic method and aid the surveillance and epidemiological study of the Ebola Virus.

One Health approach has immense potential to improve human, animal, and environmental health and combat future global health crises by creating collaborative processes connecting expertise. As a paradigm, One Health needs to involve a broad transdisciplinary effort working locally, nationally, and globally. The strategy are being discussed in our Research Topic by Tucker et al. and Yasmeen et al. Through analysis and comparison between African Swine Fever (ASF) and subsequently emerged COVID-19 pandemic, Tucker et al. pointed out that both pandemics highlight the difficulties in adequately preparing for and containing an outbreak in the face of complicated social and political factors. There are temporal and thematic links, such as similar patterns in these two threats and factors associated with ASF that compounded the COVID-19 pandemic. Moreover, two pandemics likely had asymmetric effects on each other, many of which are difficult to quantify. These two pandemics underscore the need to use a One Health framework to overcome threats from surrounding transmission to and from wildlife, exacerbating food insecurity and bottlenecks in disease surveillance capacity.

Yasmeen et al. indicated that the concept is not widely accepted in impoverished areas where zoonotic diseases often occur due to close contact with domestic or wild animals. They raised a Pakistan perspective on how to use the One Health paradigm to confront zoonotic health threats. Firstly, the study provided an overview of Pakistan's most common zoonotic diseases. Subsequently, they listed several potential factors necessary to overcome zoonotic disease prevalence, including disease outbreak surveillance, infectious waste management, disposal of hazardous materials, food safety, vector control, and health education. Finally, the authors emphasized the collaboration of governmental agencies between and with the private sector and NGOs to adopt innovative and practical practices to deal with zoonotic diseases in Pakistan.

There are some specific strategies to assist the implementation of the One Health strategy. Potential drug strategies to target vital organelles in eukaryotic cells have been identified as potential resources for novel drug design and therapy. The endoplasmic reticulum (ER) and its application as the target for drug design were summarized in reviews by Peng et al. The review clarified the role of ER stress pathways and related molecules in parasites for their survival and development, the parasitic infection-induced pathological damage in hosts and the drug resistance of parasite, which provides potential drug design targets to inhibit the development of parasites and effective treatment approaches for anti-parasite drugs.

The increasing microbial drug resistance has been the most extensive public health in recent years. Biofilms are complex microbial microcolonies of planktonic and dormant bacteria bound to a surface. Biofilm formation increases the resistance of bacteria to antibiotics and helps bacteria escape from host immune attack, which leads to clinical persistent chronic infection and other problems. Chang et al. demonstrated the advantages and challenges of bacteriophage therapy on biofilm removal, as well as the potential usage of combination therapy and genetically modified phages in a nosocomial setting, especially on artificial joint restorations and catheters. Within the One Health framework, it is crucial to combine multimodal strategies and channels to offer more access to health between humans, animals, and the environment.

In conclusion, our Research Topic focuses on the epidemiological status of primary vector-borne and zoonotic infectious diseases, the risk of pathogen spillover, novel pathogens detection system and vaccine design, concerns on One Health paradigm and application, and has generated meaningful collection in the field. Taken together, the Research Topic highlighted an urgent need to re-examine existing knowledge in the One Health approach to expand our understanding toward unraveling the underlying interplay of humans, animals, and the environment.

## Author contributions

XF contributed to the original idea and conceived the paper. XF and XZ wrote the initial draft of the paper. XF, SW, GC, XG, and XZ reviewed the final version. All authors contributed to the article and approved the submitted version.

## Funding

This work was supported in part by grants from the China Postdoctoral Science Foundation (No. 2021M692107) and the National Natural Science Foundation of China (No. 81802039).

## Conflict of interest

The authors declare that the research was conducted in the absence of any commercial or financial relationships that could be construed as a potential conflict of interest.

## Publisher's note

All claims expressed in this article are solely those of the authors and do not necessarily represent those of their affiliated organizations, or those of the publisher, the editors and the reviewers. Any product that may be evaluated in this article, or claim that may be made by its manufacturer, is not guaranteed or endorsed by the publisher.
